# Impairment of executive function in Kenyan children exposed to severe falciparum malaria with neurological involvement

**DOI:** 10.1186/1475-2875-13-365

**Published:** 2014-09-16

**Authors:** Symon M Kariuki, Amina Abubakar, Charles RJC Newton, Michael Kihara

**Affiliations:** KEMRI/Wellcome Trust Collaborative Research Programme, P.O. Box 230, 80108 Kilifi, Kenya; Nuffield Department of Medicine, University of Oxford, Oxford, UK; Department of Psychology, Lancaster University, Lancaster, UK; Department of Psychiatry, University of Oxford, Oxford, UK; Department of Psychology, United States International University, Nairobi, Kenya

**Keywords:** Children, Executive functioning, Falciparum malaria, Kenya, Acute seizures

## Abstract

**Background:**

Persistent neurocognitive impairments occur in a fifth of children hospitalized with severe falciparum malaria. There is little data on the association between different neurological phenotypes of severe malaria (seizures, impaired consciousness and prostration) and impairments in executive function.

**Methods:**

Executive functioning of children exposed to severe malaria with different neurological phenotypes (N = 58) and in those unexposed (N = 56) was examined using neuropsychological tests such as vigilance test, test for everyday attention test for children (TEA-Ch), contingency naming test (CNT) and self-ordered pointing test (SOPT). Linear regression was used to determine the association between neurological phenotypes of severe malaria and executive function performance scores, accounting for potential confounders.

**Results:**

Children with complex seizures in severe malaria performed more poorly than unexposed controls in the vigilance (median efficiency scores (interquartile range) = 4.84 (1.28-5.68) *vs.* 5.84 (4.71-6.42), P = 0.030) and SOPT (mean errors (standard deviation) = 29.50 (8.82) *vs.* 24.80 (6.50), P = 0.029) tests, but no differences were observed in TEA-Ch and CNT tests. Performance scores for other neurological phenotypes of severe malaria were similar with those of unexposed controls. After accounting for potential confounders, such as child’s age, sex, schooling; maternal age, schooling and economic activity; perinatal factors and history of seizures, complex seizures remained associated with efficiency scores in the vigilance test (beta coefficient (β) (95% confidence interval (CI)) = -0.40 (-0.67, -0.13), P = 0.006) and everyday attention scores of the TEA-Ch test (β (95% CI) = -0.57 (-1.04, -0.10), P = 0.019); the association with SOPT error scores was weak (β (95% CI) = 4.57 (-0.73-9.89), P = 0.089). Combined neurological phenotypes were not significantly associated with executive function performance scores.

**Conclusion:**

Executive function impairment in children with severe malaria is associated with specific neurological phenotypes, particularly complex seizures. Effective prophylaxis and management of malaria-associated acute seizures may improve executive functioning performance scores of children.

## Background

*Plasmodium falciparum* malaria is the most common parasitic infection for the central nervous system in endemic areas, and seizures and impaired consciousness (cerebral malaria) are the main neurological manifestations [1]. Cerebral malaria and malaria-associated seizures give rise to neurocognitive impairments, particularly in the speech, language and memory domains [2]. Persistent neurocognitive impairments have been reported in 23% of children hospitalized with falciparum malaria in rural Kenya [2]. These impairments may be related to features or phenotypes of neurological involvement (seizures, impaired consciousness and prostration) common in severe malaria [3].

It has been suggested that malaria infection produces damage to the frontal lobes and which is associated with executive function deficits. Executive functions are specific neuropsychological skills defined as self-regulatory functions responsible for organizing and directing cognitive activities, emotional response and behaviour [4]. These functions are useful in directing day-to-day activities, and play a salient role in shaping long-term outcomes e.g. scholastic achievement. Despite the salient role of executive function skills, no previous study has systematically investigated the impact of different mutually exclusive phenotypical representation of malarial disease on executive functions. The few studies looking at various aspects of executive function indicate the potential negative impact of malarial infection on executive function. For instance, a previous study in Kilifi, Kenya examined executive functioning following exposure to falciparum malaria in general, but not different phenotypes, and found that the mean scores of attention/visual search were inferior in the affected group compared to controls (42.5 vs. 45.6, p = 0.03) [5]. In another study, from a different sample in Kilifi, working memory impairment (considered a sub-domain of executive functioning) was examined in children hospitalized with falciparum malaria encephalopathy [6]. In this study, paediatric falciparum malaria was associated with impairments in recall and recognition, but not in prospective memory [6], implicating other parts of the brain other than the extended hippocampal system.

It is recognized that memory recall problems may be a reflection of retrieval deficits often classified under executive functioning, which is mainly mediated by the frontal lobe [7]. Other cognitive processes involved in executive functioning are planning, reasoning, attention and implementation of tasks [8]. Involvement of extra-hippocampal damage during malaria infection was hypothesized in a previous study [6], although it was not possible to conclude whether the observed recall impairments were a marker of executive dysfunctioning because of the tool used. The diffuse pattern of involvement found in severe falciparum malaria [9], may damage the frontal lobe resulting in impairments in executive functioning.

A number of risk factors have been associated with poor cognition: environmental and genetic factors [10], clinical factors such as coma and hypoglycaemia [11], and perinatal insults [12]. Other important risk factors of cognitive functioning include malnutrition [13] and schooling status [14]. It can be hypothesized that executive functioning in children with severe malaria is related to the specific phenotypes of malaria with neurological involvement (seizures, impaired consciousness and prostration), but which may be modified by other risk factors.

The performance of executive function in school-aged children who had been exposed to different phenotypes of severe falciparum malaria with neurological involvement was examined to characterize the patterns of executive dysfunction.

## Methods

### Study site and population

Children hospitalized to Kilifi District Hospital (KDH) with severe falciparum malaria and unexposed children selected randomly from the Kilifi Health and Demographic Surveillance System (KHDSS) database were studied. KDH is the only district level hospital in the entire KHDSS, which has a population of about 250,000 people [15]. This hospital draws most admissions from the local populations, and the commonest infectious causes of childhood admissions are malaria, bacterial and viral infections (including HIV) [16].

### Study sample and definition of terms

The study participants were 114 children aged between six and nine years, 58 of whom had been hospitalized with various malaria-related complications i.e. severe malaria with neurological involvement, and 56 unexposed children, without a history of underlying encephalopathy, who served as population controls, and were similar with the cases in terms of sex, and sociodemographic characteristics and school attendance (Table 1). The causes of hospitalization for the exposed group were severe malaria with impaired consciousness (defined as unarousable coma [Blantyre Coma Score ≤2] [17] in 30 children, complex symptomatic seizures (defined as repetitive seizures during one illness, focal seizures and/or prolonged seizures lasting >15 minutes [18, 19] but in conscious patients) in 15, prostration (defined as inability to sit, drink or breastfeed; but conscious) in 9 and seizures with fever in 4. The hospitalized children had been managed using the national and WHO guidelines [20].

**Table 1:** **The distribution of sociodemographic and medical history characteristics among those exposed to severe malaria and unexposed controls**

Characteristic	Exposed (N = 58)	Unexposed (N = 56)	Comparison
Median age in years (IQR)	6.0 (6.0-7.0)	6.0 (6.0-8.0)	Z = -1.72, P = 0.085
Male sex (%)	27 (46.6)	27 (48.2)	*X* ^2^ = 0.03, P = 0.859
Home delivery (%)	43 (81.1)	48/54 (88.9)	*X* ^2^ = 1.27, P = 0.261
Perinatal problems (%)	15 (25.8)	11 (19.1)	X = 0.63, P = 0.429
Incomplete immunization (%)	40/41 (97.6)	35/36 (97.2)	P = 1.00^a^
Median maternal age in years (IQR)	34.0 (29.0-44.0)	32.0 (27.5-37.5)	Z = -0.86, P = 0.390
Mother did not attend school (%)	29/52 (54.7)	28/53 (53.9)	*X* ^2^ = 0.01, P = 0.929
Unschooled dad (%)	14/51 (27.5)	11/49 (22.5)	*X* ^2^ = 0.33, P = 0.564
Lack of maternal economic activity (%)	19/51 (37.3)	24/51 (47.1)	*X* ^2^ = 1.01, P = 0.316
Lack of paternal economic activity (%)	12/48 (25.0)	12/46 (26.1)	*X* ^2^ = 0.02, P = 0.904
Child attends school (%)	39 (67.2)	40 (71.4)	*X* ^2^ = 0.33, P = 0.564
Median number of previous hospitalization, IQR (%)	2 (1–3)	2 (1–3)	Z = 1.70, P = 0.088
History of seizures (%)	6/54 (11.1)	7/53 (13.2)	*X* ^2^ = 0.11, P = 0.740

^a^Fisher’s exact test; IQR = interquartile range.

### Assessment of neurocognition

Children were invited for neuropsychological assessments 1–2 years after discharge from KDH, where they had been treated for severe malaria [21]. Sociodemographic and medical history information was collected during assessments. All children (those exposed and unexposed to falciparum malaria) were assessed for cognitive performance on the following domains: working memory, attention (including sustained attention), executive function (including planning), and vigilance. Working memory and attention were assessed with the *self-ordered pointing test* and the *vigilance* (a computerized visual search) *test*. Executive functions or planning ability was tested using the *contingency naming test*, while sustained attention was tested using *score* or test for everyday attention for children (TEA-Ch).

### Assessment procedure

The assessment scales used in study are well-standardized neuropsychological tests, which were culturally adapted, and their psychometric properties evaluated and have been observed to retain good psychometric properties [22].

#### Self-ordered pointing test (SOPT)

The test requires non-spatial executive abilities in order to organize and carry out and monitor a sequence of responses [23]. The SOPT is a non-verbal test involving three trials of increasing difficulty, with each trial comprising drawings of locally available materials such as drums, taps and animals [22]. In each trial the order of the arrangement of the drawings is changed. The participants were instructed in the local language (Giriama), to point to a particular drawing from a group of 8, 10, or 12, and the number of errors made was noted. The participants are required to identify all the drawings during all the three trials, by touching each once. No feedback is given to the child during the test, but they were reminded to point a different place if they kept pointing to the same drawing in each trial. A six-drawings, trial test was presented to the patient before the actual trials for practice, to ensure that the instructions are well understood. All children completed all the tests in a fixed order. The main outcome is usually the total number of errors observed in all the three group-items (8, 10 or 12) during the three trials.

#### Contingency naming test (CNT)

CNT is comprised of four trials in which one names two shapes (with one small embedded inside the big one) and colours [24]. The test is sensitive and useful in testing the function of the frontal lobe i.e. speed of retrieving names and mental set shifting with changing rules/instructions [24]. The first two trials are simple as they involve automatic naming of colours, both of which requires making of a decision under a contingency rule in the third and fourth trial. In trial 1, children were asked to name the colours of the shapes while in trial 2, the child names the external shape. In trial 3, which is a switching task, children were asked to name the colour of the shape if the internal and external shapes matched and to name the external shape if the shapes didn’t match. Finally, in trial 4 children retained the rules of trial 3 but the rules were reversed i.e. if the internal and external shapes match, the shape was to be named and if they don’t, the colour was to be named. Due to the complexity of the task, total errors, time and efficiency was computed for the first three tasks separately and then again for all the four tasks.

#### Score/ test for everyday attention test for children (TEA-Ch)

Score is a measure of a child’s everyday attention utilizing auditory attention [7]. Children were asked to place beads repetitively on a designated place every time a tone was heard from a15-minute audio tape. The consisted of 10 trials each involving presentation of 9–15 identical tones lasting about 350 milliseconds, which are separated by silent inter-stimulus intervals ranging between 500 milliseconds to 5,000 milliseconds. Normally children are asked to silently count the tones, but for appropriateness in this rural Kenyan setting, children were asked to place beads in alternate sectors of an apportioned plate every time a tone was produced. Correct performance of the task is earned a point and summated for all trials at the end of the response. Children with higher response rates were assumed to have better sustained attention.

#### Computerized visual search task (Vigilance)

*Vigilance* is a computerized perceptual task requiring attention and involves an active visual for a particular object (the target) among other objects (the distracters) [25]. Children were asked to depress a button when they saw a stick figure of a man with all limbs and not depress if it was a woman or had some limbs missing. The task generated (a) errors of omission (sustained attention), when the child forgot to depress the button on the presentation of the target stimulus and (b) errors of commission (impulsivity), when the child depressed the button without the presentation of the target stimulus. Both errors were expressed as a percentage. The efficiency score was also computed taking into account the reaction speed, number of correct responses and number of errors.

### Statistical analysis

All analyses were performed using STATA (version 11, Stata Corp, TX, USA). The raw scores were non-parametric and thus the differences between the exposed and unexposed groups were performed using Mann–Whitney *U* test, but Student t-test was used for SOPT whose scores were parametric. The differences in the proportion of categorical variables between the exposed and unexposed groups were computed using Pearson’s Chi-Square test and Fisher’s exact where appropriate.

Performance scores for all tests except SOPT were non-parametric and were thus square root-transformed to achieve normality. The univariate analysis of the association between different neurological phenotypes of severe malaria and the scores for executive function tests was determined using linear regression. The multivariate analysis of the association between different phenotypes of severe malaria and the scores for executive function tests was determined using a linear regression model with child’s age, sex, schooling; and maternal schooling and economic activity; perinatal adverse events, previous hospitalizations and history of seizures as potential confounders. Interaction between phenotypes of severe malaria and adverse perinatal events with regards to poor executive functioning scores was tested *a priori* to determine whether associations should be stratified by presence or absence of perinatal events. A two-tailed p-value of ≤0.05 is considered significant for this analysis.

## Results

All background social demographic characteristics and medical history were similar for the children exposed to neurological phenotypes of severe malaria, and those unexposed (Table 1).

### Comparison between combined exposed group and controls

Overall there was no difference in performance scores between the combined neurological phenotypes and controls in all the four tests: the *Vigilance* test, TEA-Ch, SOPT and CNT (Table 2). In a linear regression model adjusted for potential confounders, combined neurological phenotypes were weakly associated with errors of commission in the vigilance test (beta coefficient (β) (95% confidence interval (CI)) = -0.06 (-0.00-0.13), P = 0.057), but no significant associations were observed for other tests. There was no evidence of interaction between adverse perinatal events and neurological phenotypes of severe malaria with regards to performance scores for all tests, thus associations are presented without stratifying by adverse perinatal events.

**Table 2:** **The differences in median scores of vigilance test and TEA-Ch test among the four exposed-groups, and between exposed and unexposed controls**

Description	Cases				Controls	Comparison ^a^	Comparison ^b^
	Cerebral malaria	Malarial seizures	Malarial prostration	Malarial seizures with fever	Unexposed		
**Vigilance test**							
Omission^c^	0.63 (0.45-0.71)	0.46 (0.32-0.66)	0.66 (0.64-0.86)	0.71 (0.47-0.87	0.64 (0.50-0.75	*X* ^2^ = 6.08, P = 0.108	Z = 1.13, P = 0.259
Commission^c^	0.40 (0.28-0.50)	0.60 (0.37-0.78)	0.37 (0.32-0.46)	0.53 (0.30-0.63)	0.35 (0.18-0.49)	*X* ^2^ = 7.10, P = 0.069	Z = -1.22, P = 0.222
Average time	11.5 (10.9- 12.5)	9.6 (7.7-11.9)	11.6 (10.7-11.9)	11.2 (9.9-11.7)	11.6 (10.6-12.4)	*X* ^2^ = 8.351, P = 0.039	Z = 0.50, P = 0.616
Efficiency	5.6 (5.1-6.1)	4.9 (2.3-5.7)	5.9 (5.5-6.3)	4.7 (4.3-5.5)	5.9 (4.8-6.8)	*X* ^2^ = 6.265, P = 0.099	Z = 0.93, P = 0.351
**Test of everyday attention for children (TEA-Ch) test**							
Median test total (IQR)	5.0 (4.0-9.0)	3.5 (3.0-7.0)	7.0 (5.0-9.0)	4.0 (2.5-5.0)	7.0 (4.0-10.0)	*X* ^2^ = 4.62, P = 0.198	Z = 0.46, P = 0.644
**Contingency Naming Test (CNT)**							
Median total error1 (IQR)	0 (0–2.0)	0 (0–2.0)	0 (0–1.0)	0 (0–0.5)	0 (0–2.0)	*X* ^2^ = 1.57, P = 0.667	Z = 1.03, P = 0.303
Median efficiency1 (IQR)	1.5 (0.8-1.9)	1.0 (0.7-1.4)	1.3 (1.2-1.9)	1.4 (1.2-1.7)	1.3 (0.8-1.8)	*X* ^2^ = 3.70, P = 0.295	Z = -1.08, P = 0.279
Median total error2 (IQR)	1.0 (0–4.0)	2.0 (0–6.0)	1.0 (0–3.0)	0 (0.9.5)	1.0 (0–3.5)	*X* ^2^ = 1.27, P = 0.735	Z = 0.20, P = 844
Median efficiency2 (IQR)	0.5 (0.2-0.7)	0.3 (0.1-0.6)	0.5 (0.4-0.7)	0.7 (0.4-0.8)	0.5 (0.2-0.8)	*X* ^2^ = 2.86, P = 0.413	Z = -0.61, P = 0.539
Median total error3 (IQR)	12.0 (9.0-15)	13 (9.0-16.0)	11.0 (10.0-16.0)	13.5 (10.0-17.0)	12.0 (9.0-15.0)	*X* ^2^ = 0.35, P = 0.950	Z = -0.09, P = 0.927
Median efficiency3 (IQR)	0.09 (0.07-0.14)	0.10 (0.06-0.12)	0.11 (0.09-0.12)	0.09 (0.07-0.15)	0.10 (0.07-0.13)	*X* ^2^ = 1.33, P = 0.721	Z = -0.68, P = 0.499
Median total error4 (IQR)	14.0 (12.0-15.0)	14.0 (12.0-16.0)	14.0 (12.0-15.0)	15.5 (13.5-17.0)	13.0 (12.0-15.0)	*X* ^2^ = 1.45, P = 0.694	Z = -0.62, P = 0.534
Median efficiency4 (IQR)	0.05 (0.04-0.07)	0.04 (0.03-0.06)	0.05 (0.04-0.06)	0.05 (0.04-0.06)	0.05 (0.04-0.06)	*X* ^2^ = 1.05, P = 0.790	Z = -0.75, P = 0.456
**Self-ordered pointing test (SOPT)**							
Total errors	23.0 (19.0-31.0)	32.0 (22.0-35.0)	26.0 (25.0-29.0)	28.0 (24.0-36.0)	24.0 (18.0-29.0)	*X* ^2^ = 3.86, P = 0.277	t = -1.44, P = 0.152

^a^Comparison among the four groups of cases using Kruskal-Wallis test; ^b^Comparison between the combined exposed groups and unexposed controls was done using Mann–Whitney *U* test, but student *t*-test for SOPT; ^c^Proportion of participants who made errors of either omission or commission.

### Comparison between specific neurological phenotypes of severe malaria and controls

**Figure 1:**
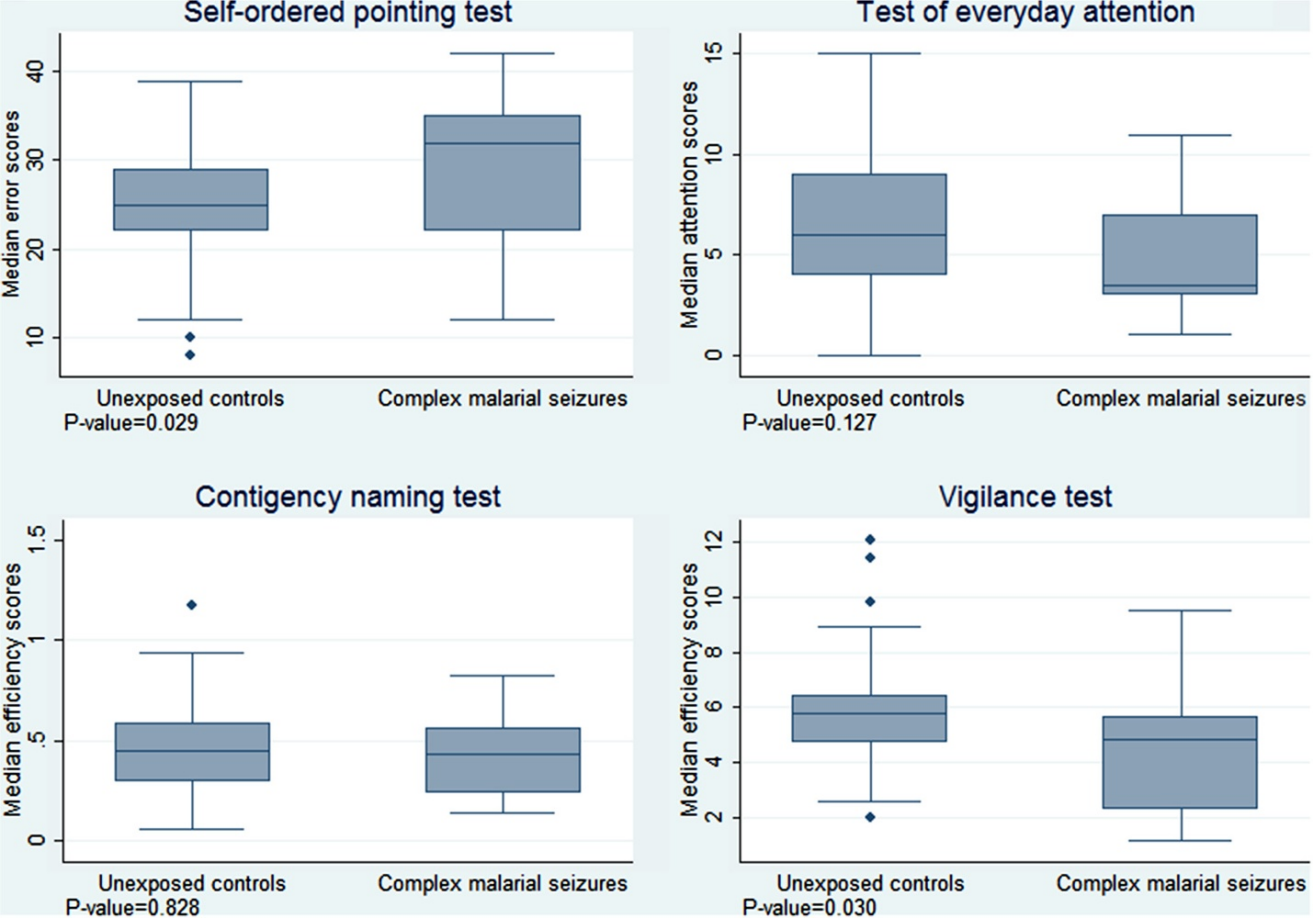
**Comparison of executive function performance scores of complex seizures in severe malaria with those of unexposed controls.** Performance scores for Vigilance test and Self-Ordered Pointing Test (SOPT) were significantly poorer for those with malarial seizures than those for unexposed controls. The differences in scores between groups were measured with Mann–Whitney *U* test for all tests, except SOPT for which Student *t*-test was used.

The *Vigilance test* efficiency scores were higher in the exposed group than controls for complex seizures (z = 2.17, P = 0.030) (Figure 1), but not for impaired consciousness (P = 0.865), prostration (P = 0.849) and seizures with fever (P = 0.223). Based on linear regression, complex seizures remained associated with efficiency scores in the multivariate analysis, which accounted for potential confounders (β (95% (CI) = -0.40 (-0.67, -0.13), P = 0.030) (Table 3).

**Table 3:** **Univariate and multivariate analysis of the association between different neurological phenotypes of severe malaria and scores for executive functioning tests in children**

Phenotypes of severe malaria	Vigilance efficiency scores, β (95% CI)	TEA-Ch test total scores, β (95% CI)	Contingency naming test average total error scores, β (95% CI)	Contingency naming test average efficiency scores, β (95% CI)	Self-ordered pointing test, β (95% CI)
Impaired consciousness: unadjusted model	0.02 (-0.12-0.18); P = 0.734	0.09 (-0.33-0.51); P = 0.667	-0.07 (-0.28-0.14); P = 0.494	0.07 (-0.02-0.16); P = 0.110	0.47 (-2.73-3.67); P = 0.771
Impaired consciousness: adjusted model	0.03 (-0.14-0.20); P = 0.756	-0.40 (-0.81-0.01); P = 0.058	0.08 (-0.35-0.19); P = 0.542	0.06 (-0.06-0.18); P = 0.329	0.99 (-3.12-5.11); P = 0.628
Complex seizures: unadjusted model	-0.35 (-0.59--0.10); P = 0.006	-0.27 (-0.81-0.27); P = 0.318	-0.04 (-0.36-0.28); P = 0.808	-0.01 (-0.12-0.12); P = 0.919	4.70 (0.50-8.90); P = 0.029
Complex seizures: adjusted model	-0.47 (-0.67--0.13); P = 0.006	-0.57 (-1.04--0.10); P = 0.019	-0.59 (-0.20-0.08); P = 0.411	0.10 (-0.32-0.52); P = 0.629	4.57 (-0.73-9.89); P = 0.089
Seizures with fever: unadjusted model	-0.16 (-0.53-0.21);P = 0.377	-0.44 (-1.40-0.52); P = 0.361	0.07 (-0.46-0.60); P = 0.802	0.09 (-0.10-0.27); P = 0.342	5.20 (-1.64-12.05); P = 0.133
Seizures with fever: adjusted model	-0.06 (-0.50-0.37); P = 0.763	-0.42 (-1.34-0.50); P = 0.352	0.18 (-0.62-0.98); P = 0.647	0.04 (-0.21-0.29); P = 0.746	2.72 (-6.07-2.59); P = 0.529
Prostration: unadjusted model	-0.00 (-0.25-0.25); P = 0.994	0.33 (-0.35-1.01); P = 0.339	-0.05 (-0.42-0.33); P = 0.800	0.10 (-0.03-0.22); P = 0.142	1.31 (-3.19-5.82); P = 0.562
Prostration: adjusted model	-0.01 (-0.26-0.25); P = 0.963	0.21 (-0.38-0.81); P = 0.474	-0.11 (-0.62-0.39); P = 0.653	0.10 (-0.06-0.25); P = 0.205	1.59 (-3.86-7.05); P = 0.555
All phenotypes: unadjusted model	-0.08 (-0.23-0.05); P = 0.219	0.01 (-0.34-0.34); P = 0.989	-0.05 (-0.23-0.12); P = 0.575	0.06 (-0.01-0.13); P = 0.090	1.98 (-0.74-4.69); P = 0.152
All phenotypes: adjusted model	-0.13 (-0.13-0.04); P = 0.137	-0.29 (-0.66-0.07); P = 0.109	-0.03 (-0.26-0.20); P = 0.800	0.04 (-0.05-0.13); P = 0.408	2.00 (-1.28-5.29); P = 0.229

TEA = test of everyday attention; β = beta coefficient; CI = confidence interval; associations were performed using linear regression, with multivariate analysis adjusted for a child’s age, sex, schooling; maternal age, schooling and economic activity; perinatal adverse events and history of seizures.

The errors of commission scores in the *Vigilance test* were higher in the exposed group than controls for complex seizures (z = -3.337, P = 0.007), but not for impaired consciousness (P = 0.913), prostration (P = 0.865) and seizures with fever (P = 0.485). In a linear regression model accounted for potential confounders, complex seizures remained associated with errors of commission (β (95% (CI) = 0.18 (0.08-0.28), P = 0.001), but other neurological phenotypes were not [impaired consciousness (P = 0.731), prostration (P = 0.519) and seizures with fever (P = 0.816)]. Compared with the unexposed group, the errors of omission (sustained attention) scores were different for complex seizures (z = 2.29, P = 0.022), but not for impaired consciousness (P = 0.457), malaria prostration (P = 0.562) and seizures with fever (P = 0.678). However in an adjusted linear regression model, errors of omission were not associated with specific neurological phenotypes of severe malaria. The overall average time to correct response differed between the exposed and unexposed group for complex seizures (z = 2.492, P = 0.012), but not for impaired consciousness (P = 0.324), prostration (P = 0.976) and seizures with fever (P = 0.552). However in an adjusted linear regression model average time to correct response was not associated with specific neurological phenotypes of severe malaria.

The scores for errors in TEA-Ch were not different between the exposed and unexposed group for complex seizures group (, P = 0.127) (Figure 1), impaired consciousness (P = 0.771), prostration (P = 0.447), seizures with fever (P = 0.165). Based on linear regression, complex seizures were associated with attention scores of the TEA-Ch test in the adjusted analysis (β (95% (CI) = -0.57 (-1.04, -0.10), P = 0.019) (Table 3).

Compared to unexposed controls complex seizures group performed significantly more errors in SOPT (t = -2.24, P = 0.029) (Figure 1), but others did not [impaired consciousness (P = 0.771), prostration (P = 0.562), seizures with fever (P = 0.133)]. However, based on linear regression, complex seizures were weakly associated with efficiency scores in the adjusted analysis (β (95% (CI) = 4.57 (-0.73-9.89), P = 0.089)) (Table 3).

There were no differences in the average errors committed in the four CNT trials between the exposed and unexposed for all clinical sub-groups: complex seizures (P = 0.993), impaired consciousness (P = 0.801), prostration (P = 0.590) and seizures with fever (P = 0.884). Average errors were not associated with neurological phenotypes in a linear regression model adjusted for potential confounders. Again there were no differences in the average efficiency in the fours CNT trials for all clinical sub-groups compared to controls: complex seizures (P = 0.828), impaired consciousness (P = 0.196), prostration (P = 0.246) and seizures with fever (P = 0.279). In a linear regression model adjusted for potential confounders, average efficiency in the fours CNT trials were not associated with neurological phenotypes of severe malaria (Table 3).

## Discussion

This study set out to determine if exposure to different neurological phenotypes of severe malaria is associated with impairments of executive functioning in children. Children exposed to complex seizures in severe malaria made more errors of commission and omission, and were less efficient in the vigilance test, and erred more in the SOPT test compared to the unexposed group, with most of these associations remaining significant after accounting for potential confounders. These pattern of results provide initial evidence that severe malaria especially when complicated with seizures negatively impact on executive functions.

The weak association between executive function scores and the combined different phenotypes of neurological involvement, suggests that executive function is affected by specific neurological phenotypes of severe malaria and that the association in previous studies may have been explained by some of these phenotypes [5]. This supposition was examined in mutually exclusive neurological phenotypes of severe malaria, and complex seizures were particularly associated with poor scores for a number of executive function tests.

### Role of seizures in impairments of executive function

Differences in cognitive scores between the complex seizures (but not other phenotypes) and unexposed group were consistently documented with a number of tests. The significant independent association between malaria seizures and efficiency scores of the vigilance test, which assessed errors of omission (sustained attention) and commission, suggests that seizures in malaria affect sustained attention and impulsivity in children. This is further supported by the independent association between errors of commission and complex malarial seizures in this study. Other previous studies have associated seizures and attention/impulsivity problems [26], although some seizures had non-malarial aetiologies [27]. Additionally, every day attention, which is related to working memory, was worse in complex seizures than unexposed controls after accounting for potential confounders. Seizures did not appear to affect working memory in a previous study [6], which used different tools that could not investigate the effects of seizures on impulsivity and sustained and everyday attention.

Working memory may be affected by complex seizures in malaria because of the more errors observed in this group compared to unexposed children in the SOPT test. This conclusion was further supported by the trend towards significance in the adjusted analysis. A previous study [6], using a different cohort, concluded that impairments in recall (measured with a different tool) in severe malaria were not related to hippocampus, and this present study suggests possible involvement of the frontal lobe which mediates executive functions. However, neuroimaging studies were not available during the period of the study to confirm this supposition.

The mechanisms for the executive function impairment following seizures in malaria is not fully understand. Seizures may cause direct neuronal damage, compounded by the pathophysiology of impaired consciousness that may be related to the seizures and/or malaria. Therefore, the lack of an association between impaired consciousness alone and executive function scores in this study may imply that the impairments documented in the previous studies of cerebral malaria [28] may be related to seizures which occur in over 80% of children with cerebral malaria [29]. The underlying genetic predisposition or neurological impairment may also be a determining factor [30]. It is known executive function is largely mediated in the frontal lobe but neuroimaging studies to identify the affected structures are required since damage to temporal lobe may extend to the frontal region [31].

### Strengths and limitations

The strength of this study lies on multifaceted neurocognitive assessment tools that are likely to tap different components of executive functioning at the same time. Single and simple executive tests have been faulted because of their low predictive value of how one performs in another different test or in the real life situation [8]. This approach allows for more confidence in our conclusions.

Executive performance in different neurological phenotypes of malaria is provided and their different pathogenetic mechanisms can easily be related to the observed psychopathologies. The multivariate analysis is accounted for several potential confounders to ensure that the differences measured reflect as much as possible the effect of exposure to different phenotypes of severe malaria. The tests used were validated in the local population and found to possess good psychometric properties [22]. However, the use of the specific neurological phenotypes was associated with small sample sizes which could have affected the power of the study. There was no data on intelligent quotients of children in the study, but school attendance was accounted for, which like intelligence, can influence neurocognitive performance [22]. Some tests such as CNT may have been difficult to all children and thus the lack of significant differences should be interpreted carefully. Data on nutritional status was not obtained, although it was not associated with test scores in a previous validation study of these tests in the same settings [22].

In conclusion, this study confirms that complex seizures in severe falciparum malaria are associated with impairments in executive functioning, suggesting possible frontal lobe involvement. Future larger studies are required to confirm these findings, accounting for potential confounders. The impairments should be assessed with more than one test since many specific cognitive components are involved, particularly impulsivity, working memory and sustained attention.
